# 182. Piperacillin/tazobactam (P/T) versus meropenem (Mero) for treatment of extended-spectrum beta-lactamase (ESBL)-producing *E.coli* and *K. pneumoniae* bloodstream infections (BSIs)

**DOI:** 10.1093/ofid/ofad500.255

**Published:** 2023-11-27

**Authors:** Alexandra L Cox, James R Beardsley, John C Williamson, Tyler J Stone, Michael E DeWitt, Elizabeth Palavecino, Vera Luther, Alex D Taylor

**Affiliations:** Atrium Health Wake Forest Baptist, Winston-Salem, Virginia; Wake Forest University School of Medicine, Winston Salem, North Carolina; Atrium Health Wake Forest Baptist, Winston-Salem, Virginia; Wake Forest Baptist Health, Winston-Salem, North Carolina; Atrium Wake Forest Baptist Health/ Wake Forest University School of Medicine, Winston-Salem, North Carolina; Wake Forest School of Medicine, Winston Salem, North Carolina; Wake Forest University School of Medicine, Winston Salem, North Carolina; Atrium Health Wake Forest Baptist, Winston-Salem, Virginia

## Abstract

**Background:**

A recent study of Mero vs. P/T for the treatment of BSIs caused by ceftriaxone-resistant Enterobacterales, including ESBL-producing organisms, revealed discouraging outcomes for patients (pts) receiving P/T. The purpose of this study is to evaluate clinical outcomes in pts treated with P/T versus Mero for BSIs caused by ESBL-producing *E. coli* or *K. pneumoniae.*

**Methods:**

This is a single health-system, retrospective, observational study. Adult inpatients with blood cultures positive for ESBL-producing *E. coli* or *K. pneumoniae* during 2016-2020 were identified by a report from an automated broth microdilution system. Pts with an isolate susceptible to both P/T and Mero who received either P/T or Mero within 24 hours of culture collection and for a duration of least 48 hours were included. Pts who had neutropenia, expired or transitioned to comfort care within 48 hours of study drug initiation, did not have source control by day 5, had a polymicrobial infection, or had a concurrent infection caused by another gram-negative organism were excluded. Pts were screened for eligibility and then randomly selected until a target of 25 pts meeting study criteria in the Mero and the P/T groups were included. The primary outcome was all-cause mortality at 30 days. Secondary outcomes included length of hospital stay, clinical and microbiological resolution by day 5, and time to clinical and microbiological resolution.

**Results:**

Forty-nine pts met study criteria and were included after one pt was excluded from P/T group due to a polymicrobial infection. Overall, the median age was 67 [53, 76], 29 (59%) pts were female, and 21 (43%) pts were admitted to an ICU at culture draw or within 24 hours. Forty-three of the 49 (88%) infections were caused by E.coli, and 38 (78%) infections originated from a urinary source. Results of the analysis are shown in table 1 and figures A-C.

**Table 1.**
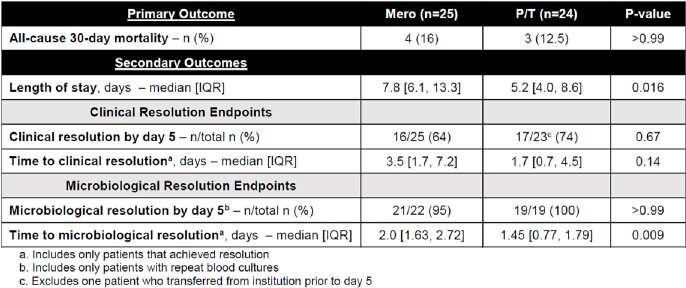
Primary and Secondary Outcomes

**Figure:**
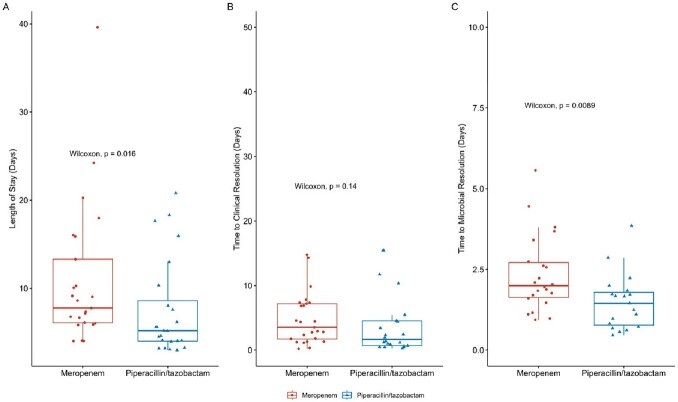
Boxplot A. Length of Stay, B. Time to Clinical Resolution, C. Time to Microbiological Resolution

**Conclusion:**

In this real-world study of pts with ESBL-producing BSIs that were predominantly from a urinary source, pts treated with P/T and Mero had similar outcomes. These results demonstrate the need for additional randomized, controlled trials, in a variety of settings to further assess outcomes in this pt population.

**Disclosures:**

**Tyler J. Stone, PharmD**, ViiV Healthcare: Employee

